# Ontogeny and programming of the fetal temporal cortical endocannabinoid system by moderate maternal nutrient reduction in baboons (*Papio* spp.)

**DOI:** 10.14814/phy2.14024

**Published:** 2019-03-25

**Authors:** Kushal Gandhi, Vanessa Montoya‐Uribe, Stacy Martinez, Samuel David, Bobby Jain, Grace Shim, Cun Li, Susan Jenkins, Peter Nathanielsz, Natalia Schlabritz‐Loutsevitch

**Affiliations:** ^1^ Department of Obstetrics and Gynecology Texas Tech University Health sciences Center at the Permian Basin Odessa Texas; ^2^ Department of Biology University of Texas at the Permian Basin Odessa Texas; ^3^ Department of Chemistry University of Texas at the Permian Basin Odessa Texas; ^4^ Department of Psychiatry Texas Tech University Health Sciences Center at the Permian Basin Odessa Texas; ^5^ University of Wyoming Laramie Wyoming; ^6^ Texas Biomedical Research Institute San Antonio Texas; ^7^ Department of Neurobiology and Pharmacology Texas Tech University Health Sciences Center Lubbock Texas

**Keywords:** Brain, endogenous cannabinoid system, fetus, maternal nutrient reduction, programming

## Abstract

Poor nutrition during pregnancy is a worldwide public health problem. Maternal nutrient reduction (MNR) is associated with maternal and fetal stress and a sex‐dependent decrease in nonhuman primate (NHP) cognitive performance. Early life stress potentiates epileptogenesis in a sex‐specific manner, and temporal lobe (TL) epilepsy is associated with neurocognitive disorders. The endogenous cannabinoid system (ECS) demonstrates remarkable developmental changes and plays a key role in aging‐related diseases (e.g., dementia). Baboons have been studied as a natural model of epilepsy and express all ECS system components. We therefore evaluated baboon fetal temporal cortex ECS ontogenic and MNR‐dependent changes. At 120 days gestational age (dGA) (term 185 days), maternal, fetal, and placental morphometry were similar between control and MNR pregnancies. MNR maternal weight gain was decreased compared with controls at 165 dGA independent of fetal sex. In male fetuses, expression of ECS synthesizing and degrading enzymes was gestational age‐dependent, with the exception of fatty acid amide hydrolase (FAAH). MNR had a sex‐specific effect on the protein expression of CB1R during development: CB1R protein expression was decreased in fetal temporal cortex of male fetuses at 120 and 140 dGA. Our data reveal that the MNR has sex‐specific effects on temporal cortical expression of the ECS in baboon offspring and shows vulnerability of ECS in male fetuses during gestation.

## Introduction

Poor nutrition during pregnancy is a worldwide public health problem (Bhutta and Das 2014; Hodgins et al. 2016; da Silva et al. 2017). Fetal tissue remodeling and growth patterns are altered by challenges in utero*, a* process termed “developmental programming”, which can have both positive and negative effects in later life (Langley‐Evans and McMullen 2010; Thornburg and Valent 2018). Maternal nutrient deficiency during pregnancy results in multiple outcomes in the offspring (Fernandez‐Twinn et al. 2005; Bautista et al. 2008; Antonow‐Schlorke et al. 2011) and affects central nervous system (CNS) structure and function (Franke et al. 2017; Ortiz‐Valladares et al. 2018). Nutrient reduction during pregnancy is associated with accelerated brain aging, altered brain structure, and decreased cognitive function in offspring demonstrated in nonhuman primate (NHP), lagomorphs, and rodents (Torres et al. 2010; Keenan et al. 2013; Reinhardt and Fanzo 2014; Huber et al. 2015; Banos‐Gomez et al. 2017; Franke et al. 2017; Illa et al. 2017; Ghaly et al. 2019; Gould et al. 2018); these effects are fetal sex‐dependent (Aiken and Ozanne 2013; Ghaly et al. 2019). The proposed mechanisms, connecting in utero conditions with altered postnatal CNS‐related function, range from control of gene expression to epigenetic and hormonal changes (Sandman et al. 2011). The imbalance between hypothalamic appetitive peptide circuits (Li et al. 2013a) and changes in glucocorticoid/leptin sensitivity in the hypothalamic‐pituitary‐adrenal (HPA) axis (Li et al. 2013b) in offspring are specifically related to maternal nutrient reduction (MNR) during pregnancy. MNR is associated with maternal and fetal stress (Huber et al. 2015; Light et al. 2018) and sex‐dependent decreases in cognitive performance have been observed in nonhuman primate (NHP) models (Rodriguez et al. 2012). Early life stress has been described to potentiate epileptogenesis in a sex‐specific manner (Huang 2014; Jones et al. 2014), and temporal lobe (TL) epilepsy is associated with neurocognitive disorders (Bostock et al. 2017).

Endocannabinoids (ECs), a family of lipid‐signaling molecules (Chan et al. 2013) and endogenous arachidonic acid (AA)‐derived mediators, are synthesized from membrane phospholipids (Alswat 2013) and are directly involved in synaptic plasticity and cortical development (Schonhofen et al. 2018). The therapeutic potential of cannabinoids in seizures is well described (Perucca 2017; Rosenberg et al. 2017). However, data on the effects of MNR on the endogenous cannabinoid system (ECS) are sparse and limited to hypothalamus, hippocampus, liver, and perirenal fat in rodent model (Ramirez‐Lopez et al. 2016a,2016b, 2017).

**Figure 1:**
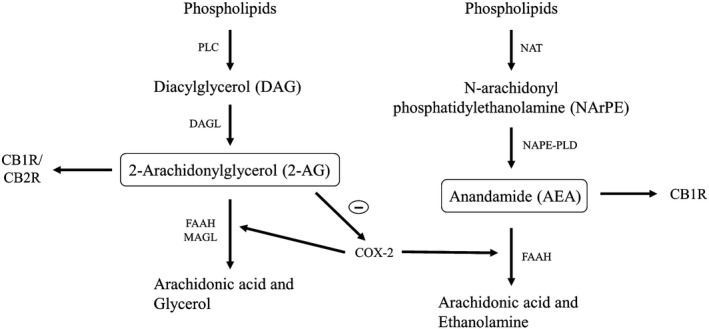
Metabolic pathways of AEA and 2‐AG synthesis, degradation and oxidation. 2‐Arachidonylglycerol (2‐AG) and Anandamide (AEA) are synthesized from phospholipids by Diacylglycerol lipase (DAGL) and N‐acyl phosphatidylethanolamine phospholipase D (NAPE‐PLD) enzymes, respectively. 2‐AG and AEA are degraded by Fatty acid amide hydrolase (FAAH), Monoacylglycerol lipase (MAGL) and Cyclooxygenase‐2 (COX‐2) enzymes into arachidonic acid. AEA mainly binds with CB1R, and 2‐AG binds with both CB1R and CB2R (endocannabinoid receptors 1 and 2). COX‐2 expression is inhibited by 2‐AG. AEA, anandamide; 2‐AG, 2‐arachidonoylglycerol; DAGL, Diacylglycerol lipase; COX‐2, cyclooxygenase‐2; FAAH, Fatty acid amide hydrolase; MAGL, Monoacylglycerol lipase; NAT, N‐Acyltransferase; NArPE, N‐Arachidonoyl phosphatidylethanolamine; PLC, Phospholipase C.

The ECS shows marked ontogenetic changes during development (Long et al. 2012; Chesworth et al. 2018) and is a key factor in aging‐related diseases (e.g., dementia) (Navarro et al. 2018). The ECS is composed of long‐chain poly‐unsaturated fatty acid (LC‐PUFA) derivatives (Torres et al. 2010), two major ligands, anandamide (AEA) and 2‐arachidonoylglycerol (2‐AG), which both bind to the cannabinoid receptor G‐protein‐coupled receptor family (CB1R and CB2R), and several synthesizing and degrading enzymes (Schlabritz‐Loutsevitch et al. 2016) (Fig. 1).

Importantly, in relation to the present study, fetal ECS is modified by changes in maternal nutritional status (Ramirez‐Lopez et al. 2016a, 2017) and pharmacological targeting of the ECS has great potential for treatment of schizophrenia (disorder, programmed by maternal folic acid deficiency (Canever et al. 2018)), autism spectrum disorder and epilepsy (Di Marzo 2018). Based on the facts that epilepsy occurs naturally in baboons (Szabo and Salinas 2016) and these NHP express all components of the ECS (Brocato et al. 2013; Rodriguez‐Sanchez et al. 2016), we sought to evaluate ontogenic and MNR‐dependent changes in the fetal temporal cortex in baboons (*Papio* spp.).

## Materials and Methods

### Animal housing and handling and the maternal nutrient reduction (MNR) model

Pregnant baboons were randomly divided into two groups: control (CTR) mothers were fed ad libitum. Starting at the 30th day of gestation, MNR mothers received 70% of the global feed consumed by the CTR group (Purina monkey diet 5038, St. Louis, MO) at the same stage of gestation. The details of animal housing and handling have been previously described (Schlabritz‐Loutsevitch et al. 2004a, 2007). All procedures were approved by the Southwest Foundation for Biomedical Research (SFBR) Institutional Animal Care and Use Committee (IACUC) and conducted in facilities approved by the Association for Assessment and Accreditation of Laboratory Animal Care (AAALAC). Fetuses were exsanguinated at C‐Section while under general anesthesia (Schlabritz‐Loutsevitch et al. 2004b). Temporal cortex tissue samples were collected immediately from fetuses of control and MNR mothers at 120, 140, and 165 days gestational age (dGA, length of gestation is 163–185 dGA (Schlabritz‐Loutsevitch et al. 2018)) and flash frozen and stored at −89°C until analyzed (Schlabritz‐Loutsevitch et al. 2007) (Table 1).

**Table 1:** Number of animals in which temporal cortex samples were obtained. Fetal male and female tissue samples from fetuses of maternal nutrient reduction (MNR) and control (CTR) baboon mothers at gestational ages of 120, 140, and 165 dGA (days of gestation)

Days gestational age (dGA)	MNR mothers		CTR mothers		TOTAL
	Male	Female	Male	Female	
120	9	3	6	4	22
140	6	3	6	6	21
165	6	6	10	10	32

### Tissue processing

#### Total RNA isolation

Total RNA was isolated from 50 to 100 mg of ground tissue samples using 1 mL of TRIzol (Cat. # 15596018 Life Technologies, USA), and the tissue was homogenized with a microtube homogenizer and vortexed for a few seconds. Each sample was phase‐separated by adding 200 *μ*L of chloroform (reagent grade S25248, CAS. No. 67‐66‐3 Fisher Science Education) followed by centrifugation at 12,000 rcf for 15 min. The top clear aqueous layer containing the RNA was aliquoted and transferred into a new 1.5‐mL tube. Total RNA was precipitated with 500 *μ*L of 100% 2‐propanol (Cat. # I9516 Sigma Life Science) at a 1:1 dilution followed by centrifugation at 12,000 rcf for 10 min, and the supernatant was discarded. The RNA pellet was washed with 1 mL of 75% ethanol (Cat. # BP2818 Fisher Scientific), centrifuged at 7500 rcf for 5 min, resuspended in 50 *μ*L of nuclease‐free water and incubated in a thermocycler at 55°C for 5–10 min. Concentrations (ng/*μ*L) of purified RNA samples were determined in a UV‐Vis spectrophotometer (R‐153 Thermo Scientific NanoDrop 2000), and the 260/280 ratio was calculated to determine sample purity. RNA samples were stored at −80°C until cDNA synthesis.

### Complementary DNA (cDNA) synthesis and quantitative reverse transcription real‐time quantitative PCR (qRT‐PCR)

Total RNA was reverse transcribed to cDNA with a Transcriptor First Strand cDNA Synthesis Kit (Cat. # 04379012001 Applied Biosystems, Roche, USA) using a final volume of 20 *μ*L per the manufacturer's instructions. qRT‐PCR was performed in triplicate in a 96‐well PCR plate using a total of 18 *μ*L per well of Fast‐Start Essential DNA Green Master Mix (Cat. # 06402712001 Applied Biosystems, Roche, USA) containing 10 *μ*L of SYBR Green I Dye, 2 *μ*L of forward primer, 2 *μ*L of reverse primer, and 4 *μ*L of nuclease‐free water. Two microliters of cDNA from the corresponding tissue samples was added for a final reaction volume of 20 *μ*L. Three negative control reactions were used: nonreverse transcription control, qRT‐PCR negative control, and Master Mix negative control. The primers 5′ (forward) GATACCACCTTCCGCACCAT and 3′ (reverse) CCGCAGTCATCTTCTCTTGGA (Millipore‐Sigma© Merck KGaA, Darmstadt, Germany) were used for quantification of *CB1R*; the primers 5′ (forward) GGAGAGGACAGAAAACAACTG and 3′ (reverse) GAGCTTGTCTAGAAGGCTTTGG were used for *CB2R*. The housekeeping gene was *β‐actin*: 5′ (forward) CCAACCGCGAGAAGATGA and 3′ (reverse) CCAGAGGCGTACAGGGATAG. The 96‐well plate was briefly centrifuged and placed in a Roche Light Cycler^®^ 96 (Applied Biosystems/Roche, USA) that was set for a three‐step reaction cycle‐assay using the time and temperature conditions shown in Table 2. Expression of *β‐actin* did not differ between the groups. Relative mean C_*T*_ values were derived are recorded, and a relative PCR quantification method (2^−ΔΔC*T*^) was performed.

**Table 2:** Maternal and fetal morphometry of control (CTR) and MNR (maternal nutrient reduction, consuming 70% of regular diet) baboons (*Papio* spp.)

	120 dGA									
	CTR Male (*n* = 6)		CTR Female (*n* = 4)		MNR Male (*n* = 9)		MNR Female (*n* = 3)		T‐test CTR versus MNR	
	Mean	SEM	Mean	SEM	Mean	SEM	Mean	SEM	Male	Female
Prepregnancy weight (kg)	17.6	1.2	14.4	0.7	15.6	1.1	16.3	1.6	0.27	0.28
Caesarian section weight (kg)	16.9	0.6	16.1	0.8	15.9	0.8	16.7	0.9	0.34	0.66
Total weight gain (kg)	−0.9	0.9	1.7	0.9	0.3	0.4	0.4	0.8	0.20	0.33
Fetal weight (g)	333.6	29.3	308.9	19.1	341.4	13.2	285.1	11.2	0.79	0.38
Fetal/maternal weight (%)	1.99	0.20	1.92	0.08	2.18	0.10	1.72	0.12	0.39	0.23
Placental weight (g)	140.8	12.9	128.1	6.9	147.3	8.9	121.0	6.1	0.68	0.49
Fetal/placental weight (%)	249.0	34.8	245.0	26.3	237.2	13.6	236.2	7.5	0.72	0.79
Brain weight (g)	40.0	2.4	38.2	1.7	39.8	1.7	35.5	1.8	0.96	0.33
Fetal brain/fetal weight (%)	12.2	0.5	12.5	0.7	11.68	0.35	12.4	0.3	0.43	0.98
Fetal brain/placental weight (%)	29.98	4.14	30.0	1.6	27.40	1.12	29.4	1.5	0.49	0.81

	140 dGA									
	CTR Male (*n* = 6)		CTR Female (*n* = 8)		MNR Male (*n* = 10)		MNR Female (*n* = 3)		T‐test CTR versus MNR	
	Mean	SEM	Mean	SEM	Mean	SEM	Mean	SEM	Male	Female
Prepregnancy weight (kg)	17.1	0.8	17.4	0.7	16.6	1.0	14.7	0.6	0.7	0.05
Caesarian section weight (kg)	18.9	0.6	18.1	0.7	18.0	1.2	15.1	0.4	0.56	0.04
Total weight gain (kg)	1.7	0.25	0.6	0.5	1.4	0.2	0.3	0.6	0.35	0.73
Fetal weight (g)	528.3	13.0	445.42	20.3	488.9	13.7	464.7	11.3	0.07	0.60
Fetal/maternal weight (%)	2.8	0.1	2.5	0.1	2.8	0.1	3.1	0.1	0.88	0.01
Placental weight (g)	181.1	16.9	159.6	7.7	159.9	5.9	156.5	3.1	0.18	0.82
Fetal/placental weight (%)	302.1	23.5	282.8	15.3	308.6	12.8	297.3	12.1	0.80	0.60
Brain weight (g)	62.43	1.66	59.83	1.29	62.97	2.70	64.51	3.67	0.90	0.15
Fetal brain/fetal weight (%)	11.8	0.3	13.6	0.4	12.9	0.3	13.90	0.7	0.05	0.70
Fetal brain/placental weight (%)	35.5	2.4	38.3	2.4	39.8	2.3	41.2	2.2	0.23	0.54

	165 dGA									
	CTR Male (*n* = 14)		CTR Female (*n* = 13)		MNR Male (*n* = 9)		MNR Female (*n* = 8)		T‐test CTR versus MNR	
	Mean	SEM	Mean	SEM	Mean	SEM	Mean	SEM	Male	Female
Prepregnancy weight (kg)	16.8	0.7	16.6	0.5	16.0	1.1	16.6	1.1	0.52	0.99
Caesarian section weight (kg)	18.7	0.7	17.9	0.5	16.1	1.1	16.8	1.1	0.055	0.30
Total weight gain (kg)	2.2	0.4	1.3	0.3	0.02	0.6	0.02	0.56	0.009	0.026
Fetal weight (g)	828.1	27.2	757.0	30.0	745.3	23.7	704.3	31.6	0.046	0.26
Fetal/maternal weight (%)	4.5	0.2	4.2	0.1	4.8	0.2	4.3	0.2	0.34	0.79
Placental weight (g)	221.4	11.8	196.0	11.8	180.3	11.1	173.0	15.2	0.027	0.27
Fetal/placental weight (%)	382.5	15.9	395.7	17.8	425.8	28.8	397.6	24.4	0.17	0.95
Brain weight (g)	85.6	2.5	79.5	2.3	81.4	2.1	77.6	1.5	0.24	0.57
Fetal brain/fetal weight (%)	9.8	0.9	10.6	0.3	11.0	0.3	11.2	0.5	0.32	0.32
Fetal brain/placental weight (%)	36.9	3.9	42.0	2.3	46.4	3.0	47.1	3.9	0.096	0.25

Note: dGA, days of gestation.

### Western blot analysis

Brain tissues were crushed in liquid nitrogen and then homogenized in RIPA buffer (Cat. # R0287, Sigma‐Aldrich, USA), 5 times at intervals of 30 sec each. Homogenates were centrifuged for 30 min at 10,000 rpm at 4°C. The supernatants were placed in new centrifuge tubes and kept at −20°C. The protein concentration per sample was quantified using a colorimetric BCA protein assay (ThermoFisher Scientific, Waltham, MA) and spectrophotometer (BioTek, Winooski, VT). The samples were warmed up at 70°C for 10 min. After that, 40 *μ*g of protein was loaded onto 10% SDS‐PAGE gels, and proteins were separated under reducing conditions and then blotted onto PVDF membranes. The membranes were blocked with 5% fat‐free milk (Cat. # 170‐6404, Bio‐Rad, USA) for 1 h. The membranes were then probed with primary antibodies to detect CB1R (1:1000; Cat. # IMG‐CB1R‐mAb001 Immunogenes, USA), CB2R (1:2000; Cat. # H00001269‐M01, Abnova, Taiwan), FAAH‐1 (1:1000; Cat. # ab54615, Abcam, USA), DAGL*α* (1:1000; Cat. # SC‐390409, Santa Cruz Biotechnology Inc., USA), MAGL (1:1000; Cat. # 100035, Cayman Chemical, Ann Arbor, MI, USA), COX‐2 (1:1000; Cat. # ab62331, Abcam, Cambridge, MA, USA), and NAPE‐PLD (1:1000; Cat. # 10305, Cayman Chemical, USA) in 1% BSA (Cat. # A4503, Sigma‐Aldrich) in TBS‐T. After washing in fresh TBS‐T (1x) (three times for a total of 30 min), the PVDF membranes were incubated with peroxidase‐conjugated anti‐mouse secondary antibody (Cat. #: 715‐035‐150, Jackson Immuno Research Laboratories, Inc., USA) and peroxidase‐conjugated anti‐rabbit secondary antibody (Cat. #: 715‐035‐152, Jackson Immuno Research Laboratories, Inc., USA) at 1:10,000 in 1% BSA in TBS‐T (1x). They were then washed in fresh TBS‐T (1x) (3 times for a total of 30 min), and the bands were detected using clarity western ECL substrate (Cat. #: 170‐5060, Bio‐Rad, USA) in a ChemiDoc‐IT TS3 815 Imager (Ultra Violet Products Ltd., UK). The PVDF membranes were kept in stripping buffer solution for 45 min at 50°C in a water bath and then washed in dH_2_O and in TBS‐T (1x) (4 times each for a total of 40 min). The membranes were blocked with 5% fat‐free milk for 1 h. PVDF membranes were probed with monoclonal anti‐*β*‐actin peroxidase antibody (1:20,000; Cat. #: A3854, Sigma, USA) in 1% BSA in TBS‐T (1x). The bands were detected using clarity western ECL substrate and a ChemiDoc‐IT TS3 815 Imager. Band intensities were quantified using ImageJ software (NIH).

### Statistical analysis

All data are presented as the mean ± standard error of the mean (SEM). *T*‐tests were used to analyze the effect of the diet (CTR vs. MNR) and fetal sex (male vs. female) in 120 dGA, 140 dGA, and 165 dGA. Treatment and age differences in gene expression and protein expression were evaluated using two‐way ANOVA and Sidak's multiple comparison test using GraphPad Prism version 6.0b software. Significance was accepted at *P* < 0.05.

## Results

### Maternal and fetal morphometry

At 120 dGA, there were no differences in maternal, fetal, or placental morphometry. The maternal weight gain in MNR mothers was less than the maternal weight gain in the CTR group at 165 dGA; this effect was independent of fetal sex (Table 2).

### *CB1R and CB2R* gene expression

*CB1R and CB2R* expression in fetal cortical tissue did not differ between CTR and MNR groups or between male and female fetuses at different days of gestation. There were no differences between the MNR and CTR groups within male and female fetuses (Table 3).

**Table 3:** Results of the ANOVA for treatment (TRT; CTR vs. MNR), gestational age (GA; 120 vs. 140 vs. 165 dGA) and the interaction between treatment and age (TRT × GA) for CB1R and CB2R gene expression in male and female fetuses

	Male fetuses		Female fetuses	
	*CB1R*	*CB2R*	*CB1R*	*CB2R*
TRT	0.74	0.35	0.87	0.36
GA	0.70	0.22	0.45	0.71
TRT x GA	0.90	0.15	0.48	0.81

### Protein expression of ECS receptors and metabolizing enzymes

In male fetuses, the expression of the synthesizing and degrading enzymes was gestational age‐dependent, with the exception of FAAH. In female fetuses, their expression was gestational age‐independent (Table 4).

**Table 4:** Results of ANOVA for treatment (TRT; CTR vs. MNR), gestational age (GA; 120 vs. 140 vs. 165 dGA) and the interaction between treatment and age (TRT × GA) for protein expression in male and female fetuses

	Male fetuses							Female fetuses						
	CB1R	CB2R	FAAH	DAGL	MAGL	COX‐2	NAPE‐PLD	CB1R	CB2R	FAAH	DAGL	MAGL	COX‐2	NAPE‐PLD
TRT	0.01	0.30	0.47	0.95	0.43	0.24	0.67	0.92	0.97	0.87	0.73	0.13	0.88	0.94
GA	0.0001	<0.0001	0.18	0.03	0.0001	0.0001	<0.0001	0.62	0.35	0.001	0.15	0.63	0.13	0.11
TRT x GA	0.049	0.45	0.92	0.88	0.88	0.70	0.97	0.68	0.69	0.53	0.67	0.005	0.79	0.95

**Figure 2:**
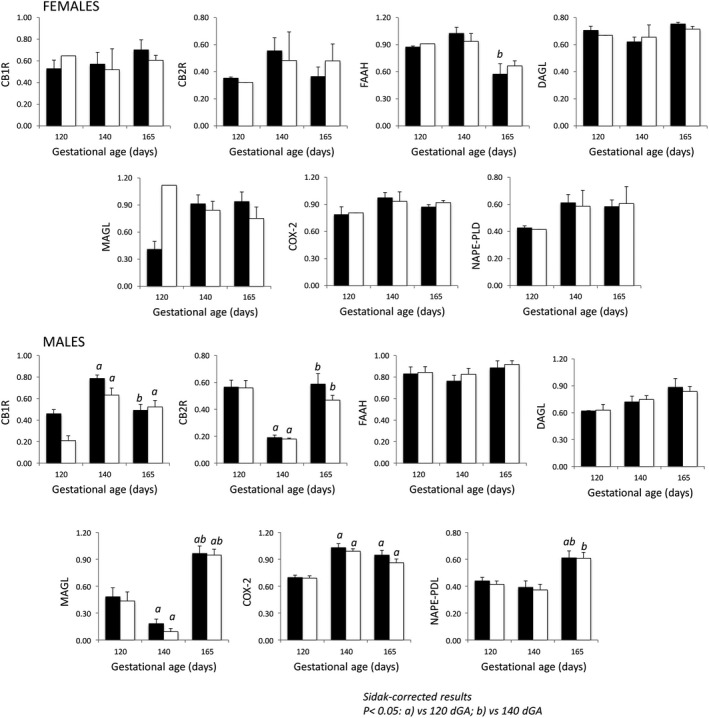
Ontogeny of the fetal cerebral endogenous cannabinoid system (ECS) CTR fed (filled) and MNR (open) male and female baboons at 120, 140 and 165 dG. Sidak‐corrected *P *< 0.05: a versus 120 dGA; b versus 140 dGA.

Ontogenic differences in ECS expression were influenced by MNR for CB1R and NAPE‐PLD proteins in male fetuses. Male fetuses exhibited ontogenic changes in the protein expression of CB1R, CB2R, MAGL, COX‐2, and NAPE‐PLD, while in the female fetuses, only FAAH demonstrated ontogenic changes (Fig. 2).

CB1R and FAAH expression levels at 165 dGA (*P* = 0.053 and 0.0026, respectively), CB2R, MAGL and NAPE‐PLD expression levels at 140 dGA (*P *=* *0.0007, *P *<* *0.0001, *P *=* *0.003, respectively) and CB1R and MAGL expression levels at 120 dGA (*P *=* *0.0013 and *P *=* *0.019, respectively) were different between male and female fetuses. Differences in FAAH and NAPE‐PLD expression levels were observed in the CTR group but not in the MNR group.

## Discussion

### Ontogenic changes in fetal cerebral ECS

Adequate nutrition is a requirement for healthy growth and development and fetal brain development is particularly sensitive to the appropriate provision of lipids, fats, vitamins, and proteins (Georgieff et al. 2018; Gould et al. 2018; Sinclair 2018). The window of fetal brain susceptibility to nutritional insult remains an open question in relation to interaction between periconceptual nutrition and offspring neurological development (Sinclair 2018). The early developmental stages (Carnegie stages 1–23) of baboon fetal development are compatible with the development of a human embryo (Hendrickx and Peterson 1997), and only the duration of the later stages is different (e.g., stage 23 corresponds to 47 dGA in baboons and 60 dGA in humans). The duration of pregnancy in baboons (163–185 dGA) is shorter than in humans (284 dGA) (Schenone et al. 2012; Schlabritz‐Loutsevitch et al. 2018). In humans, 30% of brain development takes place in utero compared to 60% in NHPs, including baboons (DeSilva and Lesnik 2008; DeSilva 2011). The CB1R receptor has been found in human brains as early as 9 weeks of gestation (63 dGA) in the subventricular zone (Biegon and Kerman 2001; Zurolo et al. 2010); this is a period of organogenesis, corresponding in the baboon to 40 dGA (Hendrickx and Peterson 1997). MNR in this study therefore started in the gestational window (30 dGA), compatible with the developmental stage of human pregnancy. The functional expression of CB1R in developing human fetal brain depends on the topography and gestational age: in the early stages of development, CB1R is expressed in the cortical plate and in neutrophils and cell stroma around mid‐gestation (Zurolo et al. 2010). In the fetal human brain, CB1R binding was found in the frontal cortex, putamen, cerebellum, and germinative subventricular zone (SVZ) from 19 to 26 weeks of gestation (Mato et al. 2003).

In general, CB1R density increases slowly in humans and in rodents with gestational age (Biegon and Kerman 2001). In our study, 140 dGA (approximately 32 weeks of human pregnancy) was associated with the higher expression of CB1R than at 120 and 165 dGA. These changes could be attributed to the growth velocity of baboon brains, which exhibit rapid growth from 125 to 140 dGA and slower growth from 140 to 165 dGA (Griffith 2012) and also – to the primate‐specific cortical expansion and gyrification in baboon fetuses (Kochunov et al. 2010). In NHPs, neuronal proliferation peaks much earlier than at 120 dGA as evaluated in this study (at approximately 90 dGA) (Rakic 2003; Kroenke et al. 2007). Comparing cortical CB1R expression in this study with data published in rodents and other species, one should take into consideration that cortical expansion in the temporal area is lower than in the parietal, medial, occipital, and lateral frontal regions in baboon fetuses (Kroenke et al. 2007). Therefore, changes in CB1R expression are specific to location, for example, CB1R increases gradually in the cortex and cerebellum but decreases in the corticospinal tract (Anavi‐Goffer and Mulder 2009; Diaz‐Alonso et al. 2012; Laprairie et al. 2012). Sex‐dependent changes in CB1R have been observed in rodent models similar to results in our study (Dow‐Edwards et al. 2016). In our study, the CB1R and CB2R protein expression levels were lower at 140 dGA than at 120 dGA and then returned to the 120 dGA level at 165 dGA. These data are in line with reports of decreased cerebral CB2R expression during gestation (Zurolo et al. 2010). CB2R is present on microglial cells, astrocytes, and on some neuron populations (Onaivi et al. 2006; Fernandez‐Ruiz et al. 2007). Microglial cells contribute to brain development in a sexually dysmorphic manner (Nelson et al. 2018), and the glial: neuron cell ratio plays a key role in brain function (Kaas 2013; Herculano‐Houzel 2014). Interestingly, in human fetuses, the number of neurons is maximal at 28 weeks of gestation and then declines by 70% around birth (Rabinowicz et al. 1996). Because the number of neurons and the glial/neuron ratio is species‐specific (Kaas 2013), the changes in CB1R and CB2R expression at approximately 140 dGA might be a result of the changes in the number of neurons: glial cells at this gestational age. MAGL and COX‐2 protein concentrations gradually increased during gestation, while changes in expression of the AEA synthesizing enzyme NAPE‐PLD were detected later in gestation in the baboon fetus. These changes might indicate a gradual decrease in cortical 2‐AG concentration in baboon fetuses because MAGL is involved in the local degradation of 2‐AG (Dinh et al. 2002; Keimpema et al. 2010). 2‐AG plays an essential role in synaptogenesis (Keimpema et al. 2010; DiPatrizio and Piomelli 2012), which in NHP picks at around 3 months of age compared to 5 years of age in humans (Bianchi et al. 2013); the 2‐AG peak has been detected immediately after birth(Maccarrone et al. 2014). Similar to our findings in the baboon, the cortical expression of NAPE‐PLD is delayed in rodents (Berghuis et al. 2007). The constant level of DAGL *α* expression in the second half of gestation found in this study is in line with the published data in rodents (Bisogno et al. 2003; Berghuis et al. 2007).

### Brain ECS response to MNR

**Figure 3:**
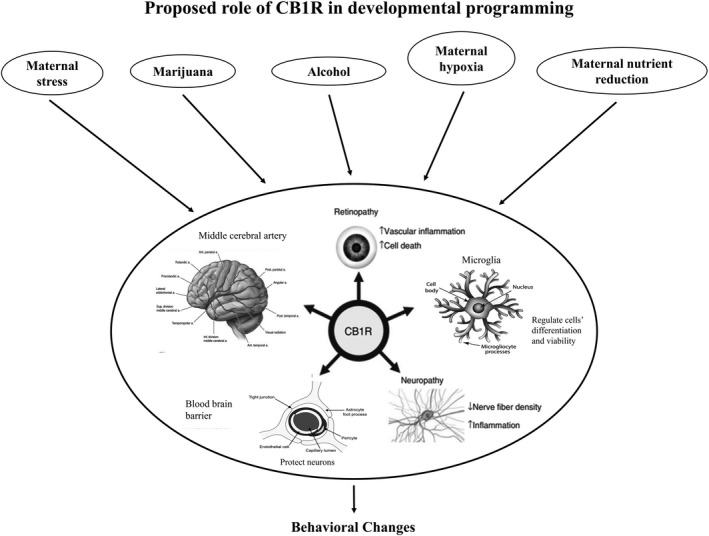
Central role of fetal cerebral CB1R expression effects of maternal stress, hypoxia, marijuana, alcohol consumption, and Maternal Nutrient Reduction on offspring behavior. Chronic stress exposure, marijuana consumption, and alcohol consumption alter EC/CB1R signaling. The ECS has been implicated in the development of several functional complications. In particular, genetic or pharmacological blockade of CB1R function has been shown to ameliorate various physiological processes associated with peripheral neuropathy, retinopathy, atherosclerosis, middle cerebral artery, and cardiac dysfunction (Walter et al. 2003; Lupica et al. 2004; Patel and Hillard 2009; Lipina et al. 2013; Seleverstov et al. 2017).

Endogenous cannabinoids are important for brain functions such as reward, cognition, learning, and memory (Mechoulam and Parker 2013). In our study, the only difference in the expression of individual components of the ECS in MNR fetuses was decreased CB1R receptor expression at 120 and 140 dGA in male fetuses. CB1R is at the center of the multiple cerebral pathways, linking maternal conditions and behavioral outcome in offspring (Fig. 3). Central role of fetal cerebral CB1R expression effects of maternal stress, hypoxia, marijuana, alcohol consumption, and maternal nutrient reduction on offspring behavior. Chronic stress exposure, marijuana consumption, and alcohol consumption alter EC/CB1R signaling. The ECS has been implicated in the development of several functional complications. In particular, genetic or pharmacological blockade of CB1R function has been shown to ameliorate various physiological processes associated with peripheral neuropathy, retinopathy, atherosclerosis, middle cerebral artery, and cardiac dysfunction in adults and in fetuses at the second half of gestation (Lupica et al. 2004; Patel and Hillard 2009; Lipina et al. 2013; Lin et al. 2015; de Salas‐Quiroga et al. 2015; Seleverstov et al. 2017).

The effect of marijuana (a CB1R agonist) resembles the effect of perinatal stress (Fride et al. 2009), and the effect of MNR on the fetal HPA axis in the baboon model has been described previously (Huber et al. 2015). Moreover, MNR induced stress‐related effects in maternal behavior in this model (Light et al. 2018). Poor maternal nutrition is associated with attention and behavior problems in offspring in the baboon (Keenan et al. 2013). CB1R is central to fetal cerebral responses to maternal alcohol consumption, maternal food intake (Coathup et al. 2017), mood disturbances, and alcohol vulnerability in offspring (Brancato et al. 2018). Maternal stress in the peri‐conceptual period is linked to infant low birth weight (Witt et al. 2016). *CB1R* expression in the hypothalamus was not affected in a rat model of MNR (Matias et al. 2003). Similar to the NHP model used in this study, offspring of the MNR group displayed a reduced weight gain and lower body weight (Ramirez‐Lopez et al. 2017). While our study did not demonstrate changes in the enzymes involved in the synthesis/degradation of ECs in opposite to a rodent model, where AA and 2‐AG levels were decreased in the hypothalamus of male and female offspring, respectively, demonstrating fetal sex‐specific effect of MNR.

A sex‐specific effect of exogenous cannabinoids on the incidence of mood disorders and cortical function has been described in rodents (Bara et al. 2018) and in humans, for example, in adolescence, the association between alcohol abuse/marijuana usage and depression is stronger in females than in males (Wilkinson et al. 2016); however, in rodents, female offspring were protected from the detrimental effect of cannabis consumption during pregnancy (Bara et al. 2018). Our data suggest that ECs undergo changes during cortical development in male, but not female, fetuses, thus providing another basis for the published data regarding sex‐specific vulnerabilities to in utero exposure to different conditions (Makinson et al. 2017; Lenz and Nelson 2018).

In conclusion, our data reveal sex‐specific developmental effects and point to a potential central role of CB1R in the offspring of MNR mothers in the second half of gestation.
